# Leukoerythroblastosis was associated with 90-day mortality in adult ICU patients with sepsis: a retrospective cohort study

**DOI:** 10.1186/s12871-026-04065-7

**Published:** 2026-07-18

**Authors:** Emi Fujii, Tomoki Tanaka, Naoto Mizumura, Yuka Segoe, Yugo Matsumoto, Mitsuhiro Fujino, Hidemitsu Miyatake, Junji Shimizu, Takuma Kishimoto, Kazunori Fujino, Yasuyuki Tsujita, Hirotoshi Kitagawa, Naoto Shiomi

**Affiliations:** 1 Department of Anesthesiology, Shiga University of Medical Science, Otsu, Japan; 2 Department of Critical and Intensive Care Medicine, Shiga University of Medical Science, Seta Tsukinowa-cho, Otsu, Shiga 520-2192 Japan; 3 Shiga University of Medical Science Emergency and Intensive Care Unit, Otsu, Japan

**Keywords:** Sepsis, Leukoerythroblastosis, Nucleated red blood cells, Immature granulocytes, Bone marrow dysfunction, Intensive care unit

## Abstract

**Background:**

Leukoerythroblastosis (LEB), defined as the concurrent detection of immature granulocytes (IG) and nucleated red blood cells (NRBC) in peripheral blood, may reflect profound bone marrow stress in sepsis. Although IG or NRBC positivity has been associated with poor outcomes, it remains uncertain whether LEB itself confers an increased risk of mortality in patients with sepsis and how its prognostic significance compares with other smear-based patterns, including isolated IG or NRBC positivity and Double-negative findings.

**Methods:**

This single-center retrospective cohort study comprised adult intensive care unit (ICU) patients with sepsis between April 2011 and March 2023. If the automated hematology analyzer generated an abnormal flag, smear microscopy was performed, and the patients were classified accordingly. The LEB group was defined as concurrent IG and NRBC on the same smear; the single-positive group included IG-only, NRBC-only, and non-concurrent both-positive findings; and the Double-negative group was defined as undetectable IG and NRBC on smear review or no smear review due to the absence of analyzer flags. To minimize immortal-time bias, we performed a day-7 landmark analysis including only patients who survived for at least 7 days after ICU admission. Associations with 90-day mortality were evaluated using Cox proportional hazards models adjusted for prespecified covariates.

**Results:**

Among 569 eligible patients, 110 (19.3%) were classified into the LEB group, 163 (28.6%) into the single-positive group, and 296 (52.0%) into the Double-negative group. The 90-day survival differed significantly among groups (log-rank *p* < 0.001), driven by worse survival in the LEB group. In the multivariable Cox model (complete-case analysis; *n* = 553; 112 events), the LEB group had higher 90-day hazard of death than the Double-negative group (adjusted hazard ratio 1.917; 95% confidence interval 1.178–3.120; *P* = 0.009), whereas the single-positive group did not differ significantly (adjusted hazard ratio 1.025; 95% confidence interval 0.629–1.672; *P* = 0.921).

**Conclusions:**

Among ICU patients with sepsis who survived for 7 days after ICU admission, LEB identified within 7 days after ICU admission was independently associated with increased 90-day mortality. Thus, LEB may reflect severe systemic stress and sepsis-associated hematopoietic dysfunction.

**Supplementary Information:**

The online version contains supplementary material available at https://doi.org/10.1186/s12871-026-04065-7.

## Background

Sepsis is defined as life-threatening organ dysfunction caused by a dysregulated host response to infection and remains a major cause of morbidity and mortality in intensive care units (ICUs) [1]. Despite advances in antimicrobial therapy and supportive care, early risk stratification remains challenging because sepsis is biologically heterogeneous and clinical deterioration can occur rapidly. Hematologic abnormalities including thrombocytopenia [2], progressive anemia [3], and lymphopenia [4], are frequently observed in patients with sepsis and have been associated with worse outcomes.

Beyond quantitative changes, increasing attention is being paid to qualitative patterns in peripheral blood assessments that may better reflect ongoing bone marrow stress and systemic inflammation. Immature granulocytes (IG) released into the circulation are considered a marker of emergency granulopoiesis and are routinely reported by modern hematology analyzers; elevated IG counts and related indices (e.g., the delta neutrophil index) are associated with sepsis severity and mortality [5–13]. Nucleated red blood cells (NRBC), typically absent in healthy adults, may appear in critical illness due to stress erythropoiesis induced by hypoxemia, inflammation, and/or bone marrow dysfunction; and are associated with adverse outcomes, including increased mortality [14–17].

However, IG and NRBC have been largely evaluated separately, and it remains unclear whether their concurrent appearance further informs risk stratification for sepsis.

Leukoerythroblastosis (LEB), the simultaneous presence of immature myeloid cells and erythroid precursors in peripheral blood, classically occurs in conditions involving profound hematopoietic stress, such as marrow infiltration, severe infection, and shock. In the ICU setting, LEB may represent a distinctive peripheral smear signature of hematopoietic stress in sepsis, potentially integrating information from both the myeloid and erythroid lineages [18, 19]. Nevertheless, only case reports and small studies specifically examined LEB in sepsis, and the clinical significance of LEB compared to single positivity (IG or NRBC alone) and double negativity (neither IG nor NRBC) has not been well characterized [20–22].

Therefore, we conducted a retrospective cohort study on adult ICU patients with sepsis to evaluate the association between LEB within 7 days after ICU admission and clinical outcomes. We defined LEB as concurrent IG and NRBC positivity on peripheral blood smear and compared the outcomes among patients with LEB, those with single-positive findings (IG-only or NRBC-only), and those with Double-negative findings. The primary outcome was the 90-day all-cause mortality. The secondary objectives were to assess the relationship between LEB and the severity of organ dysfunction and sepsis-associated hematopoietic impairment. We hypothesized that LEB identified within 7 days after ICU admission would be associated with increased 90-day mortality and greater organ dysfunction than isolated IG or NRBC positivity and Double-negative findings.

## Methods

### Study design and setting

We conducted a single-center retrospective cohort study using clinical data from adult patients admitted to the ICU of Shiga University of Medical Science Hospital, Japan, between April 1, 2011, and March 31, 2023. The study protocol was approved by the institutional ethics committee (approval No. R2025-096). The requirement for informed consent was waived because the study used anonymized patient and hospital data. Our ICU is a tertiary-care university hospital that provides advanced organ support.

### Participants

Adult ICU patients with sepsis were eligible for inclusion. Sepsis was defined according to the Sepsis-3 criteria [1], and for patients admitted before 2016, Sepsis-3 was retrospectively adjudicated from medical records. We excluded admissions for cardiovascular surgery, patients aged < 18 years, and admissions that did not meet the Sepsis-3 criteria. The primary analysis used a day-7 landmark design; therefore, patients who died within 7 days after ICU admission were excluded from the landmark cohort. We further excluded patients with hematologic malignancies or severe bone marrow disorders as well as those with missing data required for exposure classification. For patients with multiple ICU admissions, only the first admission during the study period was analyzed.

### Data collection and clinical variables

Demographics including age, sex, and body mass index (BMI), infection site, comorbidities, laboratory values, and transfusion requirements were extracted from the hospital electronic medical records and ICU information system. Blood tests were performed at ICU admission on day 1 and at approximately 6:00 AM on subsequent days. Sequential Organ Failure Assessment (SOFA) scores were calculated daily within 7 days after ICU admission. When a component could not be computed from the records, it was treated as missing. The SOFA score at ICU admission was calculated using the full SOFA score, including the central nervous system (CNS) component. For longitudinal analyses of organ dysfunction within 7 days after ICU admission and for the time-dependent Cox models, the SOFA score excluding the CNS component was used because neurological assessment may be affected by sedation during ICU care. Organ failure was defined as a SOFA subscore ≥ 2 for each organ system, and the number of failing organs was calculated daily. Continuous renal replacement therapy (CRRT) during the ICU stay was extracted from the ICU information system.

### Laboratory measurements and peripheral smear review

NRBC and IG were evaluated in the hospital laboratory. Complete blood counts (CBC) and CBC with automated differential were measured using a Sysmex XN-9000 automated hematology analyzer (Sysmex, Kobe, Japan). When CBC with automated differential testing generated abnormal analyzer flags, not limited to IG or NRBC, trained laboratory technologists performed microscopic examination of peripheral blood smears. The analyzer flag criteria used for peripheral smear preparation are summarized in Table S1. NRBC and/or IG were considered positive when observed on smear review, regardless of the absolute count. Peripheral blood smear microscopy was generally not performed in the absence of abnormal analyzer flags generated during CBC with automated differential testing. In routine laboratory practice, CBC testing itself was available 24 h a day. However, analyzer flag evaluation and peripheral smear review based on CBC with automated differential testing were performed primarily during weekday daytime hours and were generally unavailable during weekends, holidays, and nighttime hours. Therefore, CBC samples obtained outside these periods generally did not undergo analyzer flag evaluation and were not incorporated into the smear-based classification analysis.

### Exposure definition

Based on smear findings within 7 days after ICU admission, patients were classified into five groups: (1) LEB, defined as concurrent IG and NRBC positivity on the same smear; (2) non-concurrent both-positive, defined as IG and NRBC positivity on different days without concurrence; (3) NRBC-only, defined as NRBC positivity on at least one day with no IG positivity; (4) IG-only, defined as IG positivity on at least one day with no NRBC positivity; and (5) Double-negative, defined as patients in whom neither IG nor NRBC were identified on peripheral blood smear examination, or in whom smear examination was not performed within 7 days after ICU admission because no abnormal flag was generated by the automated hematology analyzer. For the primary three-group analysis, the NRBC-only, IG-only, and non-concurrent both-positive groups were combined into a single-positive group.

For dose–response analyses, an “LEB-positive day” was defined as a day on which both IG and NRBC were concurrently positive on smear review. The frequency of LEB was calculated as the total number of LEB-positive days within 7 days after ICU admission and categorized as 0, 1, 2, or ≥ 3 days. If multiple smear evaluations were performed on the same day, LEB was counted once for that day.

### Outcomes

The primary outcome was the 90-day all-cause mortality after ICU admission. Secondary outcomes included the trajectories of the SOFA score and the number of failing organs within 7 days after ICU admission. We evaluated transfusion requirements during the ICU stay and daily trajectories of lymphocyte and platelet counts within 7 days after ICU admission to explore sepsis-associated hematopoietic impairment. In our ICU, red blood cell transfusion was generally administered to maintain hemoglobin ≥ 8 g/dL; however, the final decision was at the discretion of the treating physician.

### Statistical analysis

Continuous variables are reported as median (interquartile range) and categorical variables as number (percentage). Between-group comparisons were performed using the Kruskal–Wallis test for continuous variables and the chi-square test or Fisher’s exact test for categorical variables. Survival curves were estimated using the Kaplan–Meier method and compared using the log-rank test.

The primary analysis used a day-7 landmark design to minimize the immortal-time bias. Patients who survived for 7 days after ICU admission entered follow-up at day 7 and were observed until day 90 after ICU admission. Cox proportional hazards models were fitted with robust standard errors and adjusted for pre-specified covariates (age per 10 years, sex, BMI, Charlson comorbidity index, full SOFA score including the CNS component at ICU admission, lactate at ICU admission per 1 mmol/L, presence of septic shock at ICU admission, use of mechanical ventilation at ICU admission, and use of CRRT during the ICU stay). A multivariable model was used for complete-case analysis. The analytical sample size differed across the models because of variable-specific missing covariates.

In additional sensitivity analyses, a time-dependent Cox model in a counting process (start–stop) format was constructed with follow-up from ICU day 0 to day 90 using daily time-varying exposure states based on smear findings (LEB, single-positive, and Double-negative as the reference) within 7 days after ICU admission. Because smears were not performed daily, unobserved days were handled using two prespecified assumptions: (1) the last observation was carried forward until the next smear (LOCF) and (2) persistence after the first positivity (first onward). Covariate adjustment was otherwise identical to that used in the primary Cox proportional hazards models, except that SOFA score excluding the CNS component was used because the CNS score may be influenced by sedation and mechanical ventilation during ICU care, and mechanical ventilation was not included because ventilatory support may vary during the ICU stay and could represent a post-baseline variable.

All tests were two-tailed, and statistical significance was set at *p* < 0.05. All primary analyses were performed using EZR (Saitama Medical Center, Jichi Medical University, Saitama, Japan), which is a graphical user interface for R. Time-dependent Cox models were fitted using the R software (version 4.1.0).

## Results

**Fig. 1:**
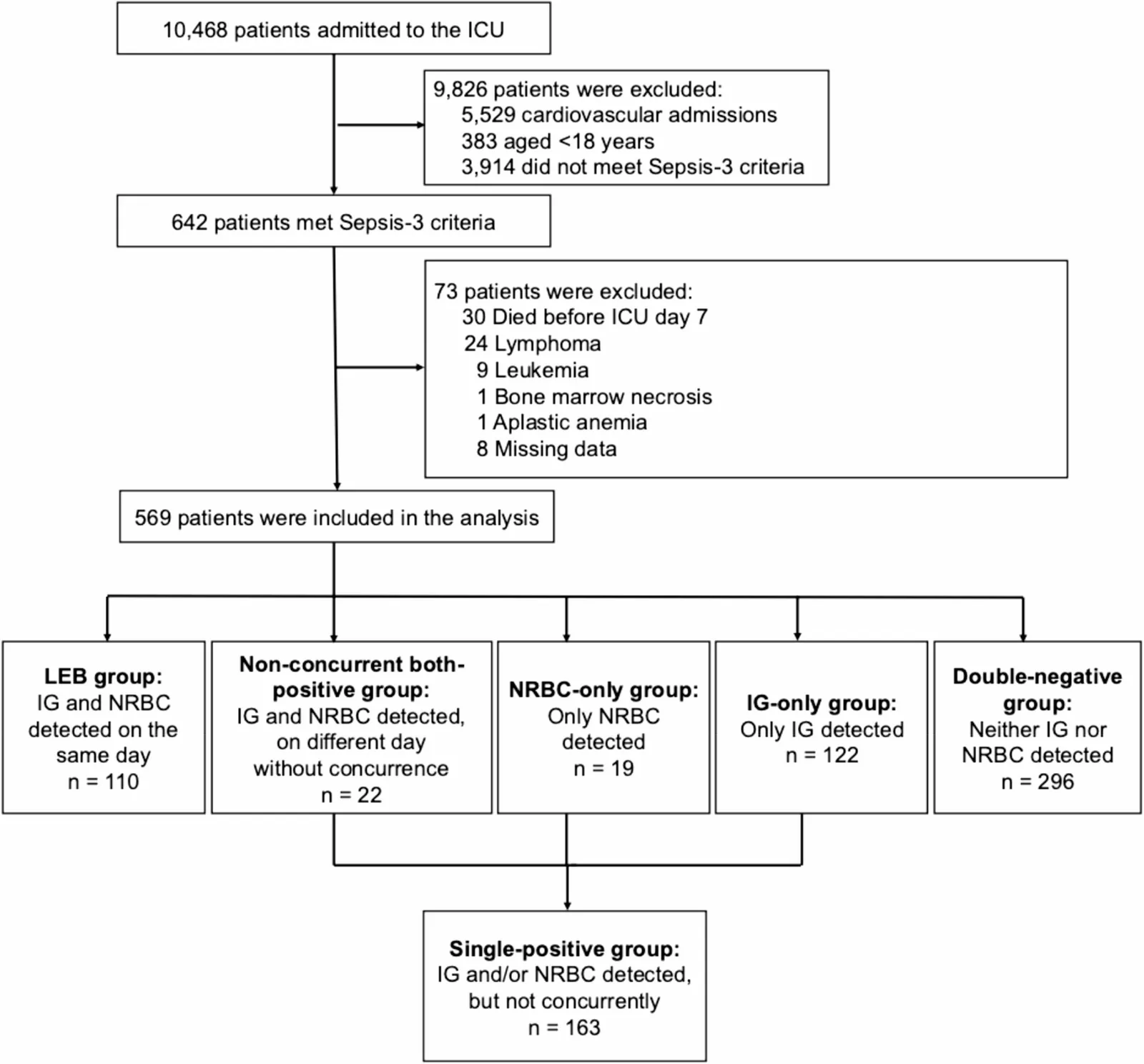
Patient selection and classification by IG and NRBC within 7 days after ICU admission. Among 10,468 ICU admissions, 569 patients meeting Sepsis-3 criteria were included after exclusions. Patients were classified into five groups according to peripheral blood smear findings within 7 days after ICU admission: LEB, defined as concurrent IG and NRBC positivity on the same smear; non-concurrent both-positive, defined as IG and NRBC positivity on different days without concurrence; NRBC-only, defined as NRBC positivity on at least one day with no IG positivity; IG-only, defined as IG positivity on at least one day with no NRBC positivity; and Double-negative, defined as patients in whom neither IG nor NRBC was identified on peripheral blood smear examination, or in whom smear examination was not performed within 7 days after ICU admission because no abnormal flag was generated by the automated hematology analyzer. For three-group analyses, the non-concurrent both-positive, NRBC-only, and IG-only groups were combined as the single-positive group. ICU, intensive care unit; IG, immature granulocytes; NRBC, nucleated red blood cells; LEB, leukoerythroblastosis

During the study period, 10,468 ICU patients were included. After exclusions, 642 adult ICU patients met Sepsis-3 criteria. Among them, 73 patients were excluded (30 died before ICU day 7, 24 had lymphoma, 9 had leukemia, 1 had bone marrow necrosis, 1 had aplastic anemia, and 8 had missing data), and the remaining 569 patients were included in the day-7 landmark cohort (Fig. 1). The baseline characteristics of patients excluded due to death within 7 days and comparisons with the included patients are summarized in Supplementary Table S2. In the day-7 landmark cohort, 110 (19.3%), 163 (28.6%), and 296 (52.0%) patients were classified into the LEB, single-positive, and Double-negative groups, respectively The frequency of CBC with automated differential tests included in the smear-based classification analysis within 7 days after ICU admission is shown in Figure S1. Fulfillment of both LEB and single-positive criteria was most frequently observed during the early period after ICU admission (Figure S2). Among patients classified as LEB and single-positive, 4 of 110 and 15 of 163 patients, respectively, first fulfilled the classification criteria only after ICU discharge.

Baseline characteristics are shown in Table 1. Overall, illness severity differed significantly among the three groups. In particular, compared with the Double-negative group, the LEB group had higher Acute Physiology and Chronic Health Evaluation (APACHE) II and SOFA score and higher lactate levels at ICU admission (all *p* < 0.001). The prevalence of septic shock and the use of CRRT and mechanical ventilation differed across groups (all *p* < 0.001). The distribution of CRRT initiation timing after ICU admission is shown in Supplementary Figure S3. ICU stay was longest in the LEB group (*p* < 0.001), and chronic steroid use was also more frequent in the LEB group (*p* = 0.01), whereas age, sex, BMI, infection site, and most comorbidities were generally similar across all the groups.

**Table 1:**
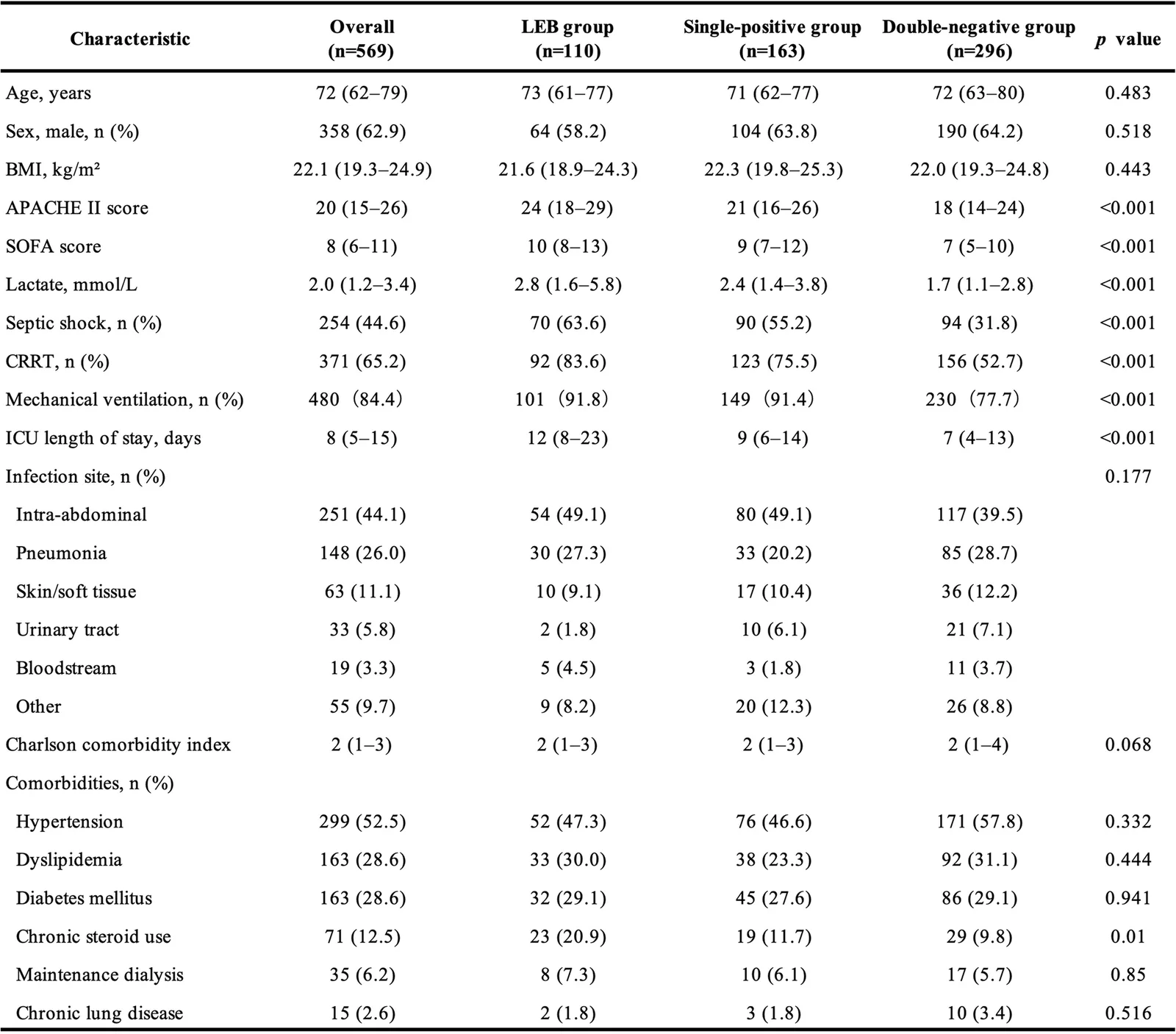
Baseline characteristics of the day-7 landmark cohort bysmear-classified groups

**Fig. 2:**
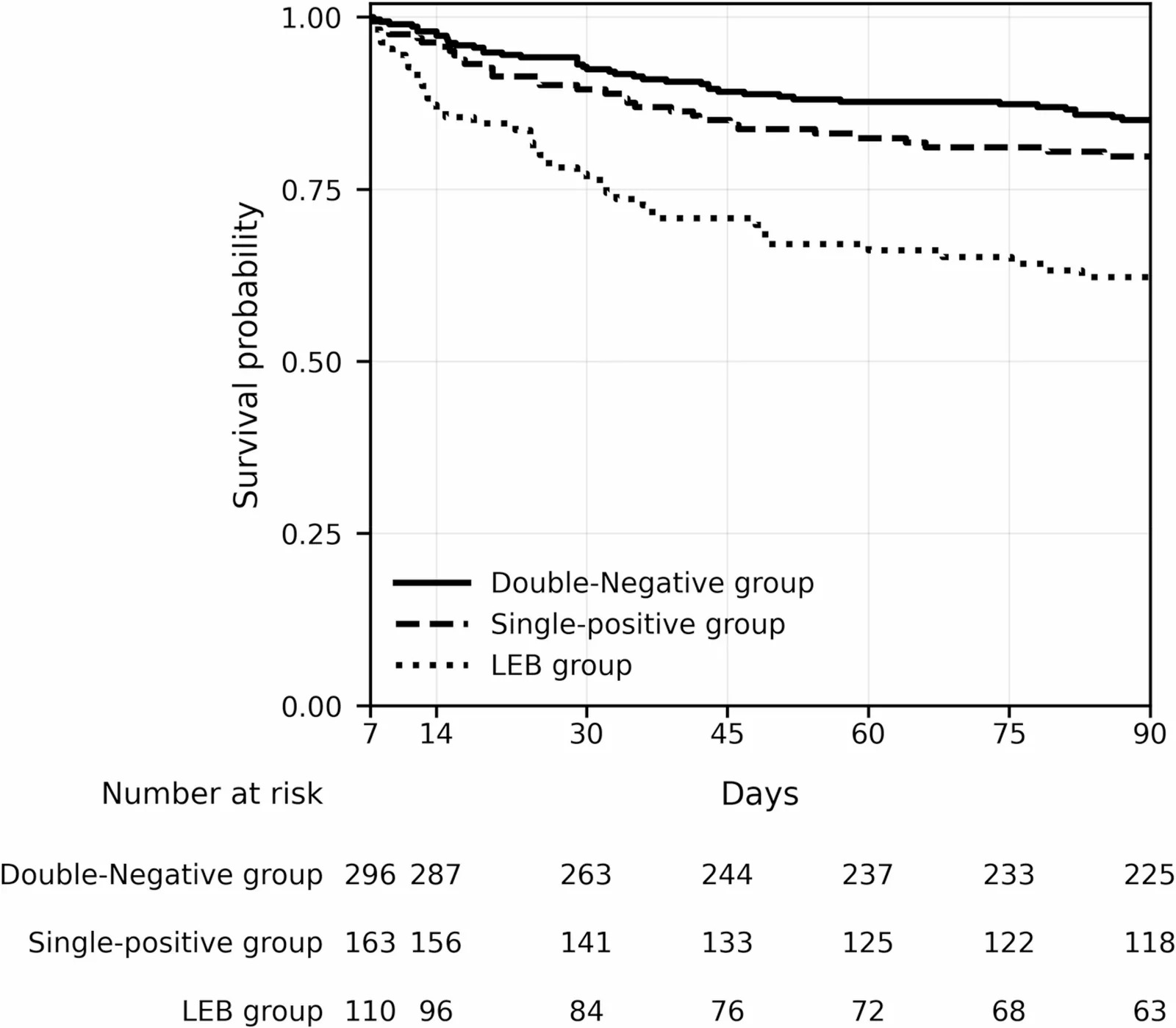
Kaplan–Meier 90-day survival in the day-7 landmark cohort according to the smear-classified groups. Patients who survived for 7 days after ICU admission were included in the study. Time zero was defined as 7 days after ICU admission (day-7 landmark), and patients were followed up until day 90 after ICU admission (censored at day 90). The groups were defined as described in Fig. 1. Numbers at risk are shown below the plot, and survival curves differed among the groups (log-rank p < 0.001). ICU, intensive care unit; LEB, leukoerythroblastosis

In the three groups (LEB, single-positive, and Double-negative; *n* = 569), 90-day survival differed significantly (log-rank *p* < 0.001; Fig. 2). The LEB group had the lowest survival, followed by the single-positive group, while the Double-negative group had the highest survival.

In Cox proportional hazards models using a day-7 landmark design with follow-up starting 7 days after ICU admission and continuing through day 90 (Table 2), the LEB group had a higher 90-day hazard of death than the Double-negative group in the multivariable model (complete-case analysis; *n* = 553; 112 events; adjusted HR 1.917, 95% CI 1.178–3.120; *P* = 0.009). Missing values among adjustment variables were limited to lactate levels (*n* = 12) and BMI (*n* = 4). In contrast, the single-positive group did not differ significantly from the Double-negative group (adjusted HR 1.025, 95% CI 0.629–1.672; *P* = 0.921). Among covariates, older age, receipt of CRRT, and use of mechanical ventilation at ICU admission were associated with an increased hazard of death, whereas higher BMI was associated with a lower hazard of death.

**Table 2:**
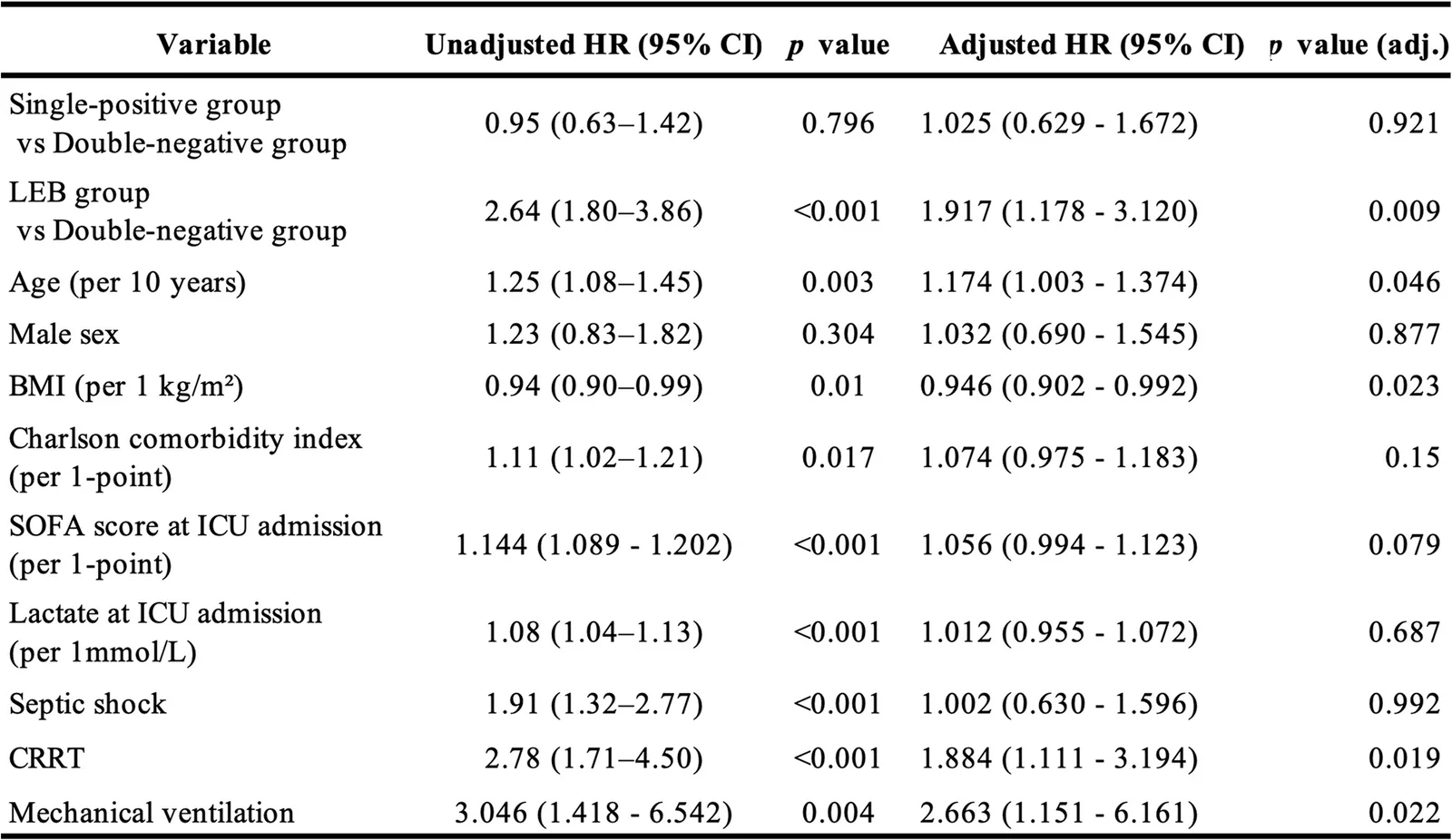
Cox proportional hazards models for 90-day mortality in the day-7 landmark cohort

**Fig. 3:**
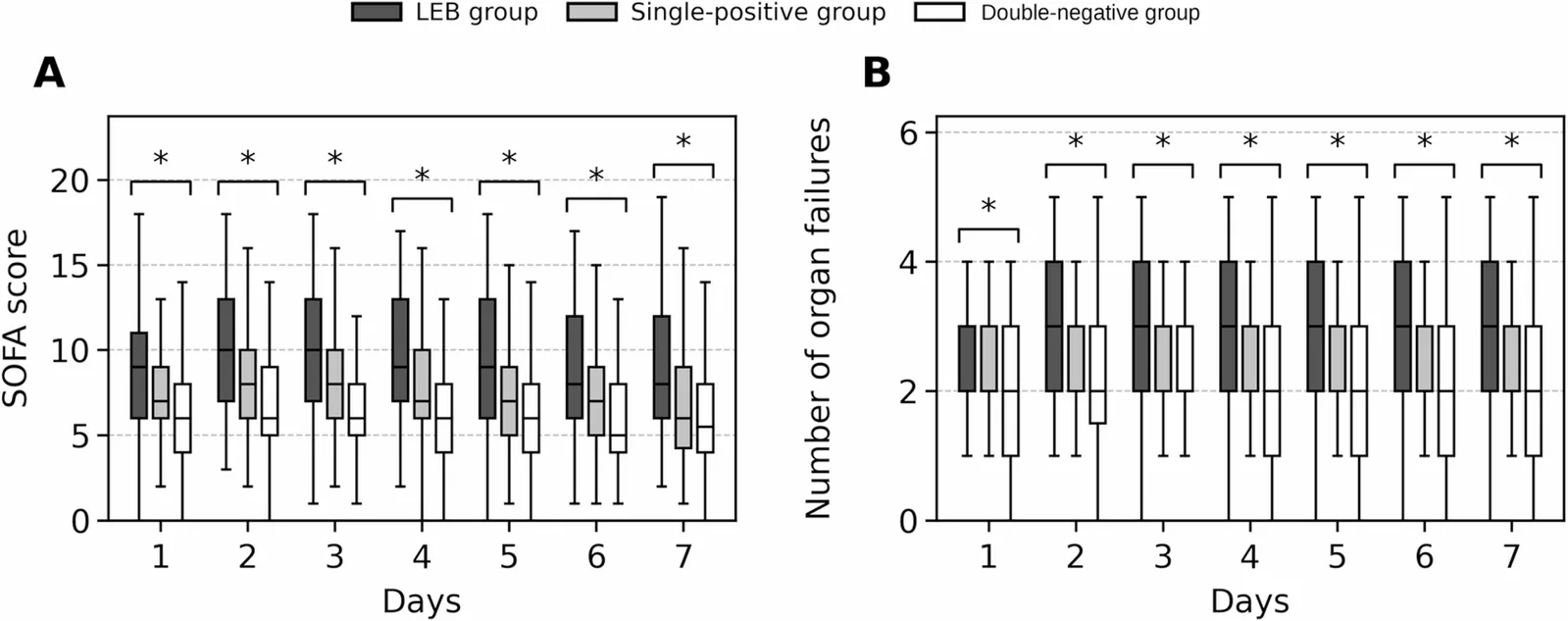
Trajectories of organ dysfunction within 7 days after ICU admission according to the smear-classified group. **A** SOFA score excluding the central nervous system component within 7 days after ICU admission. **B **Number of organ failures within 7 days after ICU admission. Box plots show the median (center line), interquartile range (box), and whiskers extending to 1.5× the interquartile range. For each day, comparisons among the three groups were performed using the Kruskal–Wallis test; *p < 0.05. ICU, intensive care unit; LEB, leukoerythroblastosis; SOFA, Sequential Organ Failure Assessment

Within 7 days after ICU admission, the LEB group had more severe organ dysfunction than the other groups. The SOFA scores (excluding the CNS) remained high throughout this period (Fig. 3A), and the number of failing organs was also high (Fig. 3B).

**Fig. 4:**
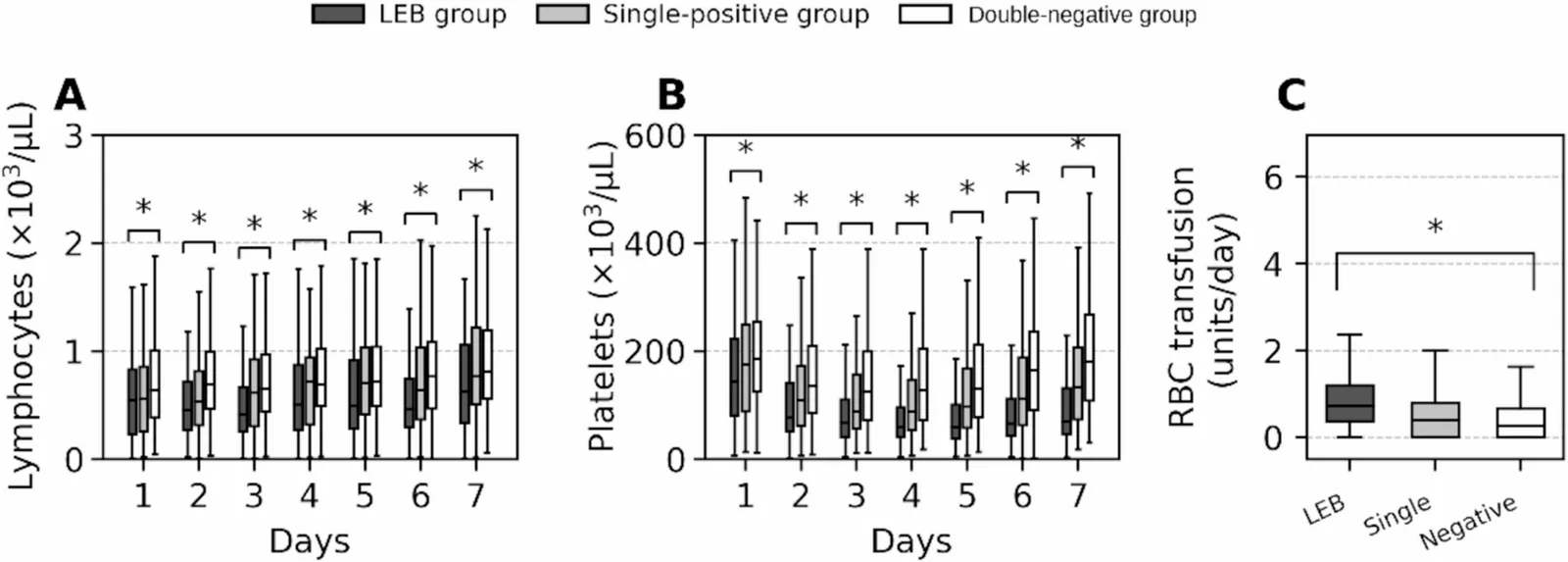
Hematologic trajectories and RBC transfusion within 7 days after ICU admission according to the smear-classified groups. **A **Lymphocyte counts within 7 days after ICU admission. **B **Platelet counts within 7 days after ICU admission. **C **Daily RBC transfusion requirements within 7 days after ICU admission. Box plots show the median (center line), interquartile range (box), and whiskers extending to 1.5× the interquartile range. For each day, comparisons among the three groups were performed using the Kruskal–Wallis test; *p < 0.05. ICU, intensive care unit; LEB, leukoerythroblastosis; RBC, red blood cell

Hematologic trajectories differed across the groups. The LEB group had lower lymphocyte and platelet counts throughout the 7 days after ICU admission (Figs. 4A and 4B) and required more red blood cell transfusions within 7 days after ICU admission (Fig. 4C).

**Fig. 5:**
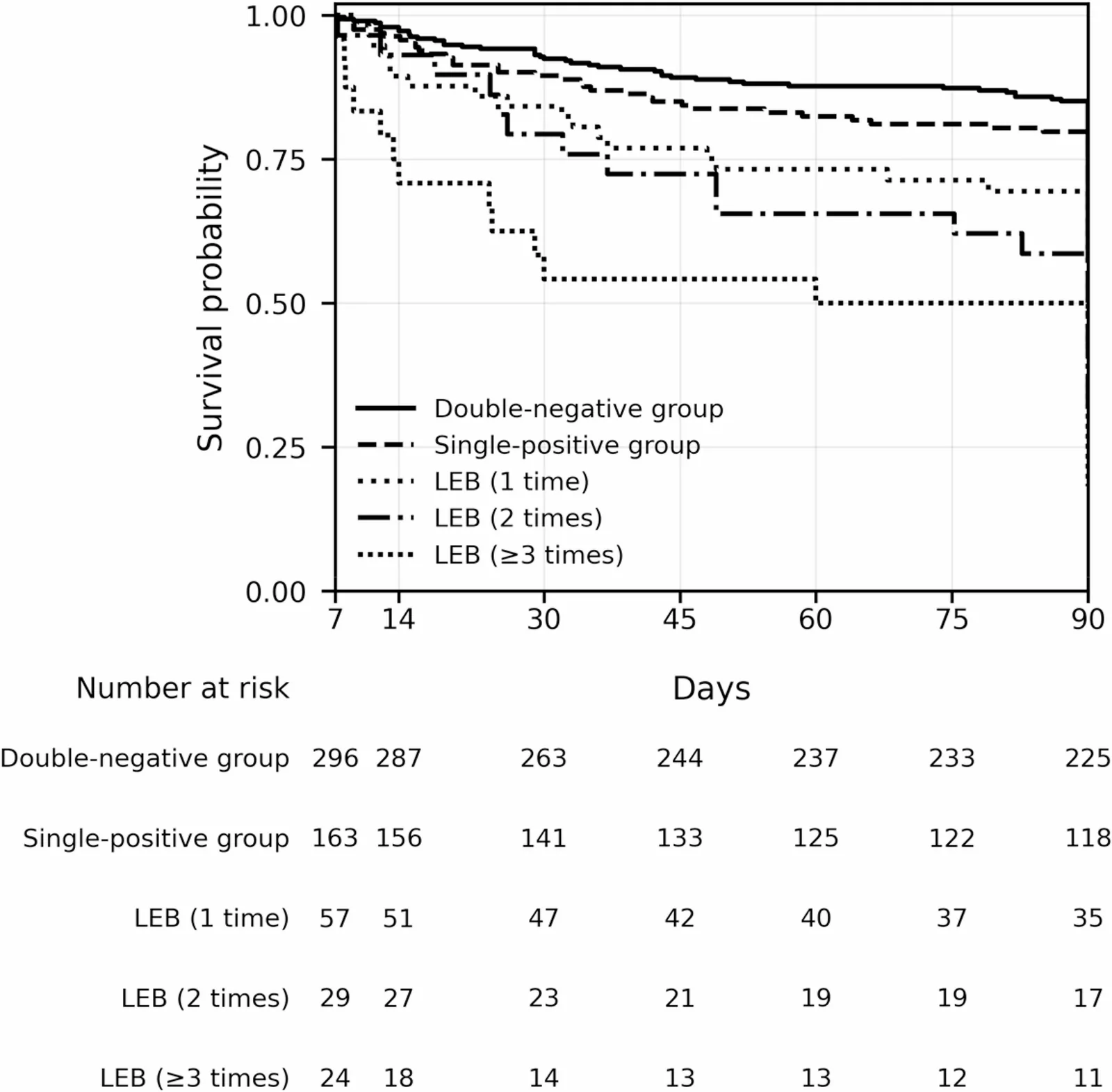
Kaplan–Meier curves for 90-day survival according to the number of LEB-positive days within 7 days after ICU admission. Kaplan–Meier curves showing 90-day survival among patients who survived for at least 7 days after ICU admission, stratified by the number of LEB-positive days within 7 days after ICU admission. Patients were categorized into five groups: Double-negative group, Single-positive group, LEB detected on 1 day, LEB detected on 2 days, and LEB detected on ≥ 3 days. Patients without any LEB-positive day were further separated into the Double-negative and single-positive groups. Survival probability decreased stepwise with increasing frequency of LEB positivity (log-rank p < 0.001). Numbers at risk are shown below the figure. LEB, leukoerythroblastosis; ICU, intensive care unit

In the landmark cohort, the 90-day survival decreased stepwise as the number of LEB-positive days within 7 days after ICU admission increased (Fig. 5; log-rank *p* < 0.001).

In the Cox model treating the number of LEB days as a continuous variable, each additional LEB-positive day was associated with a higher mortality risk, consistent with the primary landmark analysis (Table S3).

In time-dependent Cox models incorporating daily smear status as a time-varying exposure, LEB remained associated with an increased risk of death under both pre-specified assumptions for unobserved days (LOCF and first onward) (Table S4). The time-dependent analysis started from ICU day 0 and therefore included patients who did not survive until day 7; accordingly, the analytic sample size and number of events differed from the day-7 landmark cohort.

In an additional sensitivity analysis adjusting for the number of CBC with automated differential tests within 7 days after ICU admission to account for potential surveillance bias, the association between LEB and 90-day mortality remained materially unchanged (Table S5).

The results were also consistent in the sensitivity analyses using the five-group classification (LEB, non-concurrent both-positive, NRBC-only, IG-only, and Double-negative); however, the estimates in smaller subgroups were less precise because of the limited sample size (Tables S6 and S7, Figure S4).

## Discussion

In this single-center cohort of ICU patients with sepsis who survived for at least 7 days after ICU admission, LEB identified within 7 days after ICU admission was associated with worse 90-day survival. This association remained significant after multivariable adjustment in the day-7 landmark analysis, whereas the single-positive group was not independently associated with mortality. In addition, mortality increased stepwise with a higher number of LEB-positive days within 7 days after ICU admission, suggesting a dose–response relationship. Compared with the single-positive group, LEB-positive patients had more severe organ dysfunction and required more red blood cell transfusions during the early ICU course, suggesting that concurrent IG and NRBC positivity may be associated with a more severe systemic phenotype.

NRBC or IG alone can be associated with illness severity and adverse outcomes in critically ill patients, including those with sepsis [5–9, 14, 16, 17]. However, evidence specifically evaluating the prognostic significance of concurrent appearance (i.e., LEB) in a cohort setting remains limited. Our findings suggested that the simultaneous emergence of IG and NRBC identifies a high-risk subgroup, potentially reflecting more severe systemic stress than either abnormality alone. As this study was observational, LEB should be interpreted as a risk marker (or surrogate for the underlying pathophysiology) rather than a causal determinant of poor outcomes.

In the present study, LEB-positive patients developed multiple organ dysfunction more frequently, which supports the concept that bone marrow involvement may occur as part of sepsis-related organ injury. Severe systemic inflammation has been suggested to disrupt the bone marrow microenvironment, including stromal and endothelial components, and to alter hematopoietic output. Such changes could promote emergency myelopoiesis and stress erythropoiesis, leading to the premature release of myeloid precursors and nucleated erythroid cells into the peripheral circulation [23]. From this perspective, we speculate that LEB may serve as a readily observable peripheral signature of bone marrow stress or dysfunction during severe sepsis and septic shock [24].

Consistent with this interpretation, LEB was accompanied by thrombocytopenia, persistent lymphopenia, and progressive anemia, requiring more red blood cell transfusions within 7 days after ICU admission [2–4]. Sepsis-associated thrombocytopenia is a multifactorial phenomenon that reflects both consumption and impaired production, and impaired thrombopoiesis related to bone marrow dysfunction may be present in some patients [25]. Sustained anemia and increased transfusion needs may likewise reflect inflammation-driven suppression of erythropoiesis, dysregulated iron metabolism, and/or disruption of marrow niche function [26, 27]. Experimental studies have suggested that sepsis can injure the bone marrow niche and alter stromal/endothelial support, which may, in turn, impair multilineage hematopoiesis [23]. Therefore, the concurrent appearance of IG and NRBC may represent a composite indicator of systemic inflammation and hematopoietic dysregulation.

In addition to serving as markers of severity, immature erythroid and myeloid populations may actively contribute to sepsis-induced immunosuppression. Circulating CD71 + CD235a+ erythroid cells have been reported to expand during sepsis and exhibit immunomodulatory properties [28], and increased levels have been associated with nosocomial infection and mortality [29]. Immature neutrophil subsets have also been implicated in impaired antimicrobial responses and dysregulated inflammation [30–32]. Collectively, these findings suggest that LEB may reflect an immunologically vulnerable state associated with severe systemic stress and adverse outcomes.

Peripheral smear findings are inexpensive, widely available, and require no additional blood sampling. If externally validated, early identification of LEB in the ICU may help clinicians identify patients at higher risk, guide closer monitoring, and anticipate complications such as secondary infections and persistent organ dysfunction. Future studies should determine whether smear-based findings provide additional clinically useful prognostic information and whether temporal changes in LEB reflect treatment response or recovery.

This study had several limitations. First, because we adopted a day-7 landmark design to reduce immortal-time bias, the primary analysis included only patients who survived for at least 7 days after ICU admission. Therefore, our findings should be interpreted as applying to day-7 survivors rather than to the overall population of ICU patients with sepsis. Patients who died before the landmark time point may have had different hematologic profiles and disease trajectories, potentially limiting the generalizability of our findings. Second, as this was a single-center retrospective observational study, residual confounding cannot be excluded. Third, smear microscopy review was not performed routinely; rather, it was triggered based on automated hematology analyzer flags. In routine laboratory practice, analyzer flag evaluation triggering peripheral smear review was performed only during weekday daytime hours and was unavailable during weekends, holidays, and nighttime hours. Therefore, selection bias and exposure misclassification could not be entirely excluded because smear review was not performed for all samples. In addition, detailed records regarding which samples actually underwent smear review were not retained long term in our laboratory system, precluding accurate retrospective quantification of smear review frequency. Previous studies evaluating the Sysmex XN/XR series have demonstrated good agreement between analyzer-based detection of IG or NRBC and manual smear review [33, 34]. Nevertheless, because smear review was not performed universally, exposure misclassification remains a limitation of the present study, although any resulting bias would likely have attenuated the observed associations toward the null. Fourth, time-dependent Cox analyses require assumptions for unobserved days (e.g., LOCF and first onward), and the results should be interpreted with caution. Finally, the overall rate of continuous renal replacement therapy was high, likely reflecting our tertiary care case mix and this may limit external generalizability.

## Conclusions

Among ICU patients with sepsis who survived for at least 7 days after ICU admission, leukoerythroblastosis identified within 7 days after ICU admission was independently associated with increased 90-day mortality in a day-7 landmark analysis. LEB may reflect severe systemic stress and sepsis-associated hematopoietic dysfunction.

## Supplementary Information

Additional file 1: Supplementary tables.

Additional file 2: Supplementary figures.

## Data Availability

The datasets used and/or analyzed in the current study are available from the corresponding author upon reasonable request.

## Abbreviations

APACHE: Acute Physiology and Chronic Health Evaluation
BMI: Body Mass Index
CBC: Complete Blood Count
CI: Confidence Interval
CNS: Central Nervous System
CRRT: Continuous Renal Replacement Therapy
HR: Hazard Ratio
ICU: Intensive Care Unit
IG: Immature Granulocytes
LEB: Leukoerythroblastosis
LOCF: Last Observation Carried Forward
NRBC: Nucleated Red Blood Cells
SOFA: Sequential Organ Failure Assessment
