# Supplementary material for: Leukoerythroblastosis was associated with 90-day mortality in adult ICU patients with sepsis: a retrospective cohort study
Source: BMC Anesthesiol. 2026 Jul 18;26:615. doi: 10.1186/s12871-026-04065-7 (PMC13663248; doi:10.1186/s12871-026-04065-7)
Supplement: Supplementary file 1 [file 12871_2026_4065_MOESM1_ESM.pdf]

Table S1. Analyzer flag criteria for peripheral blood smear preparation using the Sysmex XN-9000 hematology analyzer.

Peripheral blood smears were prepared when any of the following analyzer flags or threshold criteria generated by the Sysmex XN-9000 automated hematology analyzer (Sysmex, Kobe, Japan) were met. “Manufacturer-defined flagging criterion” refers to a proprietary analyzer algorithm implemented by the manufacturer for which a numerical threshold was not available in the original analyzer setting file.

Category	Flag	Smear preparation criterion
WBC	WBC Abn Scattergram	Manufacturer-defined flagging criterion
WBC	Neutropenia	NEUT# < 0.0 (10^2/uL); NEUT% < 30.0 (%)
WBC	Neutrophilia	NEUT# > 999.0 (10^2/uL); NEUT% > 90.0 (%)
WBC	Lymphocytosis	LYMPH# > 99.0 (10^2/uL); LYMPH% > 40.0 (%)
WBC	Monocytosis	MONO# >= 5,000/uL; MONO% > 13.0 (%)
WBC	Eosinophilia	EO# > 99.0 (10^2/uL); EO% > 15.0 (%)
WBC	Basophilia	BASO# >= 500/uL; BASO% > 2.0 (%)
WBC	Leukocytopenia	WBC < 2.00 (10^3/uL)
WBC	Leukocytosis	WBC > 15.00 (10^3/uL)
WBC	NRBC Present	NRBC > 1.0 (%)
WBC	IG Present	IG# > 1.0 (10^2/uL); IG% > 100.0 (%)
WBC	Blasts?	Manufacturer-defined flagging criterion
WBC	Abn Lympho?	Manufacturer-defined flagging criterion
WBC	Blasts/Abn Lympho?	Manufacturer-defined flagging criterion
WBC	Left Shift?	Manufacturer-defined flagging criterion
WBC	Atypical Lympho?	Manufacturer-defined flagging criterion
RBC	RBC Abn Distribution	Manufacturer-defined flagging criterion
RBC	Dimorphic Population	Manufacturer-defined flagging criterion
RBC	RET Abn Scattergram	Manufacturer-defined flagging criterion
RBC	Reticulocytosis	RET > 500 (10^4/uL); RET% > 5.0 (%)
RBC	Anisocytosis	RDW-SD > 99.0 fL; RDW-CV > 18.0 (%)
RBC	Microcytosis	MCV < 60.0 fL
RBC	Macrocytosis	MCV > 110.0 fL
RBC	Hypochromia	MCHC < 29.0 g/dL
RBC	Anemia	HGB < 8.0 g/dL
RBC	Erythrocytosis	RBC > 7.00 (10^6/uL)
RBC	RBC Agglutination?	Manufacturer-defined flagging criterion
RBC	Turbidity/HGB Interference?	Manufacturer-defined flagging criterion
RBC	HGB Defect?	Manufacturer-defined flagging criterion
RBC	Fragments?	Manufacturer-defined flagging criterion
PLT	PLT Abn Scattergram	Manufacturer-defined flagging criterion
PLT	PLT Abn Distribution	Manufacturer-defined flagging criterion
PLT	Thrombocytopenia	PLT < 100 (10^3/uL)
PLT	Thrombocytosis	PLT > 500 (10^3/uL)

WBC, white blood cell; RBC, red blood ce ll; PLT, platelet; NRBC, nucleated red blood cell; IG, immature granulocyte; RET, reticulocyte; HGB, hemoglobin; MCV, mean corpuscular volume; MCHC, mean corpuscular hemoglobin concentration; RDW, red cell distribution width.

Table S2. Baseline characteristics of patients included in the day-7 landmark cohort and those who died within 7 days after ICU admission

Data are presented as medians (interquartile ranges) or numbers (percentages), as appropriate. P-values were calculated using the Kruskal–Wallis test for continuous variables and the chi-square test or Fisher's exact test for categorical variables, as appropriate. Peripheral smear classification was based on smear findings within 7 days after ICU admission. The groups were defined as described in Fig. 1.

Characteristic	Included in landmark cohort (n=569)	Died before ICU day 7 (n=30)	p
Age, years	72 (62–79)	74 (62–77)	0.851
Sex, male, n (%)	358 (62.9)	17 (56.7)	
BMI, kg/m²	22.1 (19.3–24.9)	21.1 (16.8–23.2)	0.114
APACHE II score	20 (15–26)	33 (24–4)1	<0.001
SOFA score	8 (6–11)	13(11-17)	<0.001
Peripheral smear classification, n (%)			0.005
LEB group	110(19.3)	13(43.3)	
Single-positive group	163(28.6)	8(26.7)	
Double-negative group	296(52.0)	9(30.0)	
Lactate, mmol/L	2.0 (1.2–3.4)	5.0 (2.1–9.6)	<0.001
Septic shock, n (%)	254 (44.6)	24 (80.0)	<0.01
CRRT, n (%)	371 (65.2)	24 (80.0)	0.096
Mechanical ventilation, n (%)	480 (84.4)	26 (86.7)	0.734
ICU length of stay, days	8 (5–15)	2 (2–4)	<0.001
Infection site, n (%)			0.114
Intra-abdominal	251 (44.1)	8 (26.7)	
Pneumonia	148 (26.0)	8 (26.7)	
Skin/soft tissue	63 (11.1)	4 (13.0)	
Urinary tract	33 (5.8)	1 (3.3)	
Bloodstream	19 (3.3)	3 (10.0)	
Other	55 (9.7)	6 (20.0)	
Charlson comorbidity index	2 (1–3)	3 (1–5)	0.196
Comorbidities, n (%)			
Hypertension	299 (52.5)	15 (50.0)	0.968
Dyslipidemia	163 (28.6)	7 (23.3)	0.913
Diabetes mellitus	163 (28.6)	11 (36.7)	0.346
Chronic steroid use	71 (12.5)	2 (6.7)	0.343
Maintenance dialysis	35 (6.2)	5 (16.7)	0.025
Chronic lung disease	15 (2.6)	1 (3.3)	0.817

APACHE II, Acute Physiology and Chronic Health Evaluation II; SOFA, Sequential Organ Failure Assessment; ICU, intensive care unit; LEB, leukoerythroblastosis; BMI, body mass index; CRRT, continuous renal replacement therapy.

Table S3. Cox models of 90-day mortality by number of LEB days within 7 days after ICU admission

Follow-up started 7 days after ICU admission (day-7 landmark) and continued until day 90 after ICU admission (censored on day 90). Complete-case analysis was performed (n = 553; 112 events). Missing values among adjustment variables were limited to lactate levels (n = 12) and BMI (n = 4). HRs with 95% CIs were estimated using univariable and multivariable Cox proportional hazards models. The multivariable model was adjusted for all covariates listed in this table. The groups were defined as described in Fig. 1. The LEB occurrence count represents the number of LEB-positive days within 7 days after ICU admission.

Variable	Unadjusted HR (95% CI)	*p* value	Adjusted HR (95% CI)	*p* value
LEB occurrence count within 7 days (per 1 additional day)	1.575 (1.351 – 1.836)	<0.001	1.257 (1.049 - 1.507)	0.013
Age (per 10 years)	1.251 (1.080 – 1.448)	0.003	1.157 (0.991 - 1.352)	0.066
Male sex	1.228 (0.830 – 1.816)	0.304	1.002 (0.671 - 1.498)	0.991
BMI (per 1 kg/m²)	0.943 (0.902 – 0.986)	0.01	0.947 (0.903 - 0.994)	0.027
Charlson comorbidity index (per 1-point)	1.111 (1.019 – 1.210)	0.017	1.071 (0.971 - 1.182)	0.17
SOFA score at ICU admission (per 1-point)	1.144 (1.089 - 1.202)	<0.001	1.054 (0.991 - 1.122)	0.093
Lactate at ICU admission (per 1mmol/L)	1.082 (1.037 – 1.130)	<0.001	1.011 (0.954 - 1.073)	0.704
Septic shock	1.908 (1.315 – 2.767)	<0.001	1.029 (0.650 - 1.628)	0.903
CRRT	2.775 (1.713 – 4.496)	<0.001	1.903 (1.126 - 3.214)	0.016
Mechanical ventilation	3.046 (1.418 - 6.542)	0.004	2.627 (1.139 - 6.060)	0.024

BMI, body mass index; CI, confidence interval; CRRT, continuous renal replacement therapy; HR, hazard ratio; ICU, intensive care unit; LEB, leukoerythroblastosis; SOFA, Sequential Organ Failure Assessment.

Table S4. Time-dependent Cox proportional hazards models for 90-day mortality (exposure updating within 7 days after ICU admission)

The outcome was 90-day all-cause mortality. Follow-up started on ICU admission (day 0) and continued until day 90 (censored on day 90). Daily smear status was treated as a time-varying exposure and updated using smear findings within 7 days after ICU admission; for days without smear review, exposure status was imputed using two prespecified assumptions: (A) last observation carried forward (LOCF; current-status model) and (B) persistence after first positivity (first onward model). After ICU day 7, the last available exposure status was assessed at the end of the follow-up. Robust (sandwich) standard errors were used to account for within-patient correlations arising from multiple start–stop intervals per patient. The complete case analysis included 580 patients, contributing to 41,322 intervals with 137 deaths. The analytical sample differs from the day-7 landmark cohort because this model does not condition on survival to day 7 and excludes intervals with missing covariates. The groups (LEB, single-positive, and Double-negative) were defined as described in Fig. 1.

S4A. Current-status model (exposure can change daily)

Variable	Unadjusted HR (95% CI)	p value	Adjusted HR (95% CI)	p value (adj.)
Single-positive vs Double-negative	1.75 (1.08–2.82)	0.022	1.42 (0.85–2.37)	0.186
LEB vs Double-negative	4.79 (3.03–7.58)	<0.001	2.90 (1.76–4.78)	<0.001
Age (per 10 years)	1.20 (1.06–1.35)	0.003	1.10 (0.96–1.27)	0.171
Sex, male	1.09 (0.77–1.54)	0.63	0.84 (0.58–1.22)	0.366
BMI (per 1 kg/m²)	0.95 (0.90–1.00)	0.056	0.95 (0.89–1.01)	0.097
Charlson comorbidity index (per 1-point)	1.13 (1.05–1.22)	0.001	1.12 (1.02–1.23)	0.014
SOFA score at ICU admission (excluding CNS; per 1-point)	1.22 (1.16–1.29)	<0.001	1.14 (1.06–1.23)	<0.001
Lactate at ICU admission (per 1 mmol/L)	1.11 (1.06–1.16)	<0.001	1.04 (0.99–1.09)	0.162
Septic shock	2.34 (1.66–3.28)	<0.001	1.08 (0.70–1.67)	0.713
CRRT	2.84 (1.84–4.39)	<0.001	1.56 (0.92–2.64)	0.097

S4B. First-onward model (hierarchical: once Single-positive/LEB occurs, status is carried forward)

Variable	Unadjusted HR (95% CI)	p value	Adjusted HR (95% CI)	p value (adj.)
Single-positive vs Double-negative	1.63 (1.07–2.48)	0.024	1.14 (0.73–1.79)	0.566
LEB vs Double-negative	3.88 (2.59–5.81)	<0.001	2.13 (1.35–3.36)	0.001
Age (per 10 years)	1.20 (1.06–1.35)	0.003	1.11 (0.96–1.28)	0.144
Sex, male	1.09 (0.77–1.54)	0.63	0.91 (0.62–1.32)	0.609
BMI (per 1 kg/m^2)	0.95 (0.90–1.00)	0.056	0.95 (0.89–1.01)	0.091
Charlson comorbidity index (per 1-point)	1.13 (1.05–1.22)	0.001	1.11 (1.00–1.22)	0.043
SOFA score at ICU admission (excluding CNS; per 1-point)	1.22 (1.16–1.29)	<0.001	1.14 (1.05–1.22)	<0.001
Lactate at ICU admission (per 1 mmol/L)	1.11 (1.06–1.16)	<0.001	1.04 (0.98–1.09)	0.195
Septic shock	2.34 (1.66–3.28)	<0.001	1.06 (0.68–1.65)	0.795
CRRT	2.84 (1.84–4.39)	<0.001	1.51 (0.90–2.53)	0.123

CNS, central nervous system; CRRT, continuous renal replacement therapy; ICU, intensive care unit; IG, immature granulocytes; NRBC, nucleated red blood cells; SOFA, Sequential Organ Failure Assessment.

Table S5. Cox proportional hazards models for 90-day mortality additionally adjusted for the frequency of CBC with automated differential tests within 7 days after ICU admission

Follow-up started on ICU day 7 (landmark) and continued until day 90 after ICU admission (censored at day 90). HRs with 95% CIs were estimated using univariable and multivariable Cox proportional hazards models. The multivariable model was additionally adjusted for the number of CBC with automated differential tests within 7 days after ICU admission to account for potential surveillance bias related to differential opportunities for smear-based evaluation.

The Double-negative group served as the reference category for the exposure variable. Age was modeled per 10-year increase, BMI per 1 kg/m² increase, Charlson comorbidity index per 1-point increase, SOFA score per 1-point increase, lactate level per 1 mmol/L increase, and number of CBC with automated differential tests per 1-test increase.

Variable	Unadjusted HR (95% CI)	*p* value	Adjusted HR (95% CI)	*p* value
Single-positive group vs Double-negative group	0.95 (0.63–1.42)	0.796	1.068 (0.652 - 1.751)	0.794
LEB group vs Double-negative group	2.64 (1.80–3.86)	<0.001	2.013 (1.230 - 3.296)	0.005
Age (per 10 years)	1.251 (1.080 – 1.448)	0.003	1.178 (1.006 - 1.378)	0.042
Male sex	1.228 (0.830 – 1.816)	0.304	1.059 (0.706 - 1.588)	0.782
BMI (per 1 kg/m²)	0.943 (0.902 – 0.986)	0.01	0.950 (0.905 - 0.996)	0.035
Charlson comorbidity index (per 1-point)	1.111 (1.019 – 1.210)	0.017	1.072 (0.973 - 1.181)	0.16
SOFA score at ICU admission (per 1-point)	1.144 (1.089 - 1.202)	<0.001	1.055 (0.993 - 1.122)	0.085
Lactate at ICU admission (per 1mmol/L)	1.082 (1.037 – 1.130)	<0.001	1.007 (0.949 - 1.068)	0.825
Septic shock	1.908 (1.315 – 2.767)	<0.001	1.016 (0.637 - 1.622)	0.945
CRRT	2.775 (1.713 – 4.496)	<0.001	1.976 (1.157 - 3.375)	0.013
Mechanical ventilation	3.046 (1.418 - 6.542)	0.004	2.855 (1.224 - 6.659)	0.015
Number of CBC with automated differential tests	1.073 (0.871 - 1.322)	0.507	0.838 (0.662 - 1.061)	0.142

Table S6. Baseline characteristics of the day-7 landmark cohort according to the smear-classified five-group analysis

Data are presented as medians (interquartile ranges) or numbers (percentages), as appropriate. The groups were defined as described in Fig. 1. P-values were calculated using the Kruskal–Wallis test for continuous variables and the chi-square test or Fisher's exact test for categorical variables, as appropriate. SOFA score and lactate were measured at ICU admission.

Variable	LEB group (n=110)	Non-concurrent both-positive group (n=22)	IG-only group (n=122)	NRBC-only group (n=19)	Double-negative group (n=296)	*p* value
Age, years	73 (61–77)	72 (67–79)	71 (56–77)	73 (70–77)	72 (63–80)	0.21
Sex, male, n (%)	64 (58.2)	14 (63.6)	82 (67.2)	8 (42.1)	190 (64.2)	0.218
APACHE II score	24 (18–29)	26 (21–30)	20 (16–25)	24 (16–28)	18 (14–24)	<0.001
SOFA score (excluding CNS) at ICU admission	9 (7–12)	10 (8–13)	8 (6–10)	8 (7–11)	7 (5–8)	<0.001
Lactate at ICU admission, mmol/L	2.8 (1.6–5.7)	2.8 (1.9–6.2)	2.3 (1.2–3.7)	1.9 (1.1–3.2)	1.6 (1.1–2.7)	<0.001
Septic shock, n (%)	70 (63.6)	16 (72.7)	68 (55.7)	6 (31.6)	94 (31.8)	<0.001
CRRT, n (%)	92 (83.6)	17 (77.3)	90 (73.8)	16 (84.2)	156 (52.7)	<0.001
Chronic steroid use, n (%)	23 (20.9)	3 (13.6)	12 (9.8)	4 (21.1)	29 (9.8)	0.024
Mechanical ventilation, n (%)	101 (91.8)	21 (95.5)	112 (91.8)	16 (84.2)	230 (77.7)	<0.001

APACHE II, Acute Physiology and Chronic Health Evaluation II; SOFA, Sequential Organ Failure Assessment; ICU, intensive care unit; LEB, leukoerythroblastosis; NRBC, nucleated red blood cells; CRRT, continuous renal replacement therapy.

Table S7. Cox proportional hazards models for 90-day mortality in the day-7 landmark cohort (five-group classification)

Follow-up started 7 days after ICU admission (day-7 landmark) and continued until day 90 after ICU admission (censored on day 90). The groups were defined as described in Fig. 1, with the Double-negative group serving as the reference category. HRs with 95% CIs were estimated using univariable and multivariable Cox proportional hazards models. The multivariable model was adjusted for age (per 10 years), sex, body mass index, Charlson comorbidity index, SOFA score at ICU admission, lactate level at ICU admission (per 1 mmol/L), septic shock at ICU admission, CRRT during the ICU stay, and use of mechanical ventilation at ICU admission. Complete-case analysis was performed (n = 553; 112 events).

Variable	Unadjusted HR (95% CI)	*p* value	Adjusted HR (95% CI)	*p* value
**Five-group classification (reference: Double-negative group)**				
LEB group	2.64 (1.80–3.86)	<0.001	1.895 (1.161 - 3.095)	0.011
Non-concurrent both-positive group	1.52 (0.67–3.46)	0.318	1.287 (0.530 - 3.128)	0.577
NRBC-only group	0.50 (0.12–2.03)	0.327	0.222 (0.030 - 1.645)	0.141
IG-only group	0.94 (0.60–1.47)	0.773	1.145 (0.676 - 1.941)	0.615
**Covariates**				
Age (per 10 years)	1.25 (1.08–1.45)	0.003	1.182 (1.011 - 1.382)	0.036
Male sex	1.23 (0.83–1.82)	0.304	0.986 (0.658 - 1.478)	0.946
Body mass index (per 1 kg/m^2)	0.94 (0.90–0.99)	0.01	0.947 (0.903 - 0.992)	0.023
Charlson comorbidity index (per 1-point)	1.11 (1.02–1.21)	0.017	1.076 (0.977 - 1.186)	0.137
SOFA score at ICU admission (per 1-point)	1.14 (1.09 - 1.20)	<0.001	1.061 (0.997 - 1.130)	0.062
Lactate at ICU admission (per 1mmol/L)	1.08 (1.04–1.13)	<0.001	1.018 (0.961 - 1.079)	0.543
Septic shock	1.91 (1.32–2.77)	<0.001	0.930 (0.579 - 1.493)	0.764
CRRT	2.78 (1.71–4.50)	<0.001	1.917 (1.130 - 3.250)	0.016
Mechanical ventilation	3.05 (1.42 - 6.54)	0.004	2.677 (1.157 - 6.194)	0.021

BMI, body mass index; CI, confidence interval; CRRT, continuous renal replacement therapy; HR, hazard ratio; ICU, intensive care unit; IG, immature granulocytes; LEB, leukoerythroblastosis; NRBC, nucleated red blood cells; SOFA, Sequential Organ Failure Assessment.
