# Supplementary material for: Leukoerythroblastosis was associated with 90-day mortality in adult ICU patients with sepsis: a retrospective cohort study
Source: BMC Anesthesiol. 2026 Jul 18;26:615. doi: 10.1186/s12871-026-04065-7 (PMC13663248; doi:10.1186/s12871-026-04065-7)
Supplement: Supplementary file 2 [file 12871_2026_4065_MOESM2_ESM.pdf]

Figure S1. Distribution of CBC with automated differential tests included in smear-based exposure classification within 7 days after ICU admission

Histogram showing the distribution of the number of CBC with automated differential tests included in the analysis within 7 days after ICU admission. Although multiple CBC measurements could be performed clinically within the same day, only one representative CBC with automated differential test per day was included in the analysis. In routine laboratory practice, analyzer flag evaluation triggering peripheral smear review was generally available only during weekday daytime hours; therefore, not all CBC measurements were eligible for smear-based classification analysis, and the maximum number of evaluable tests within 7 days after ICU admission was five in most patients. The accompanying table shows the distribution of CBC with automated differential test frequencies according to the smear-classified groups.

CBC, complete blood count; ICU, intensive care unit; LEB, leukoerythroblastosis.

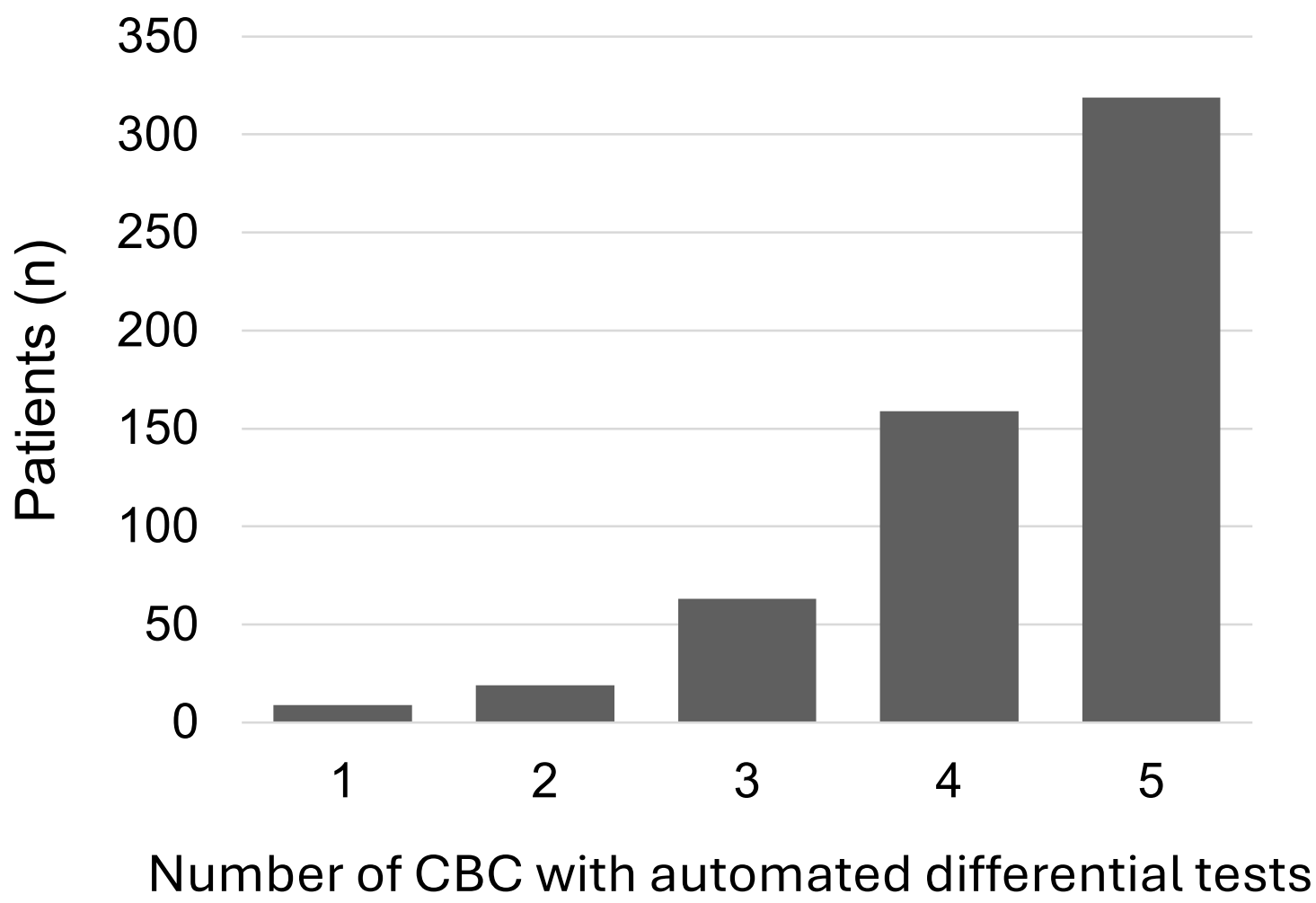

Group	Number of CBC with automated differential tests , n (%)					Total
	1	2	3	4	5	
LEB group	1 (0.9)	0 (0.0)	7 (6.4)	39 (35.5)	63 (57.3)	110
Single-positive group	1 (0.6)	1 (0.6)	10 (6.1)	42 (25.8)	109 (66.9)	163
Double-negative group	7 (2.4)	18 (6.1)	46 (15.5)	78 (26.4)	147 (49.7)	296
Total	9 (1.6)	19 (3.3)	63 (11.1)	159 (27.9)	319 (56.1)	569

Figure S2. Distribution of the first day meeting the smear-classified group criteria within 7 days after ICU admission

(A) Histogram showing the distribution of the first ICU day on which patients fulfilled the criteria for LEB (concurrent positivity for immature granulocytes [IG] and nucleated red blood cells [NRBC] on the same smear review).

(B) Histogram showing the distribution of the first ICU day on which patients fulfilled the criteria for the single-positive group (IG-only, NRBC-only, or non-concurrent positivity for both findings).

Dark gray bars indicate patients who first fulfilled the criteria within 7 days after ICU admission whereas light gray bars indicate patients who first fulfilled the criteria only after ICU discharge.

Among the patients classified as LEB, 4 of 110 first fulfilled the LEB criteria after ICU discharge. Similarly, among the patients classified as single-positive, 15 of 163 first fulfilled the respective criteria after ICU discharge.

ICU, intensive care unit; LEB, leukoerythroblastosis; IG, immature granulocytes; NRBC, nucleated red blood cells.

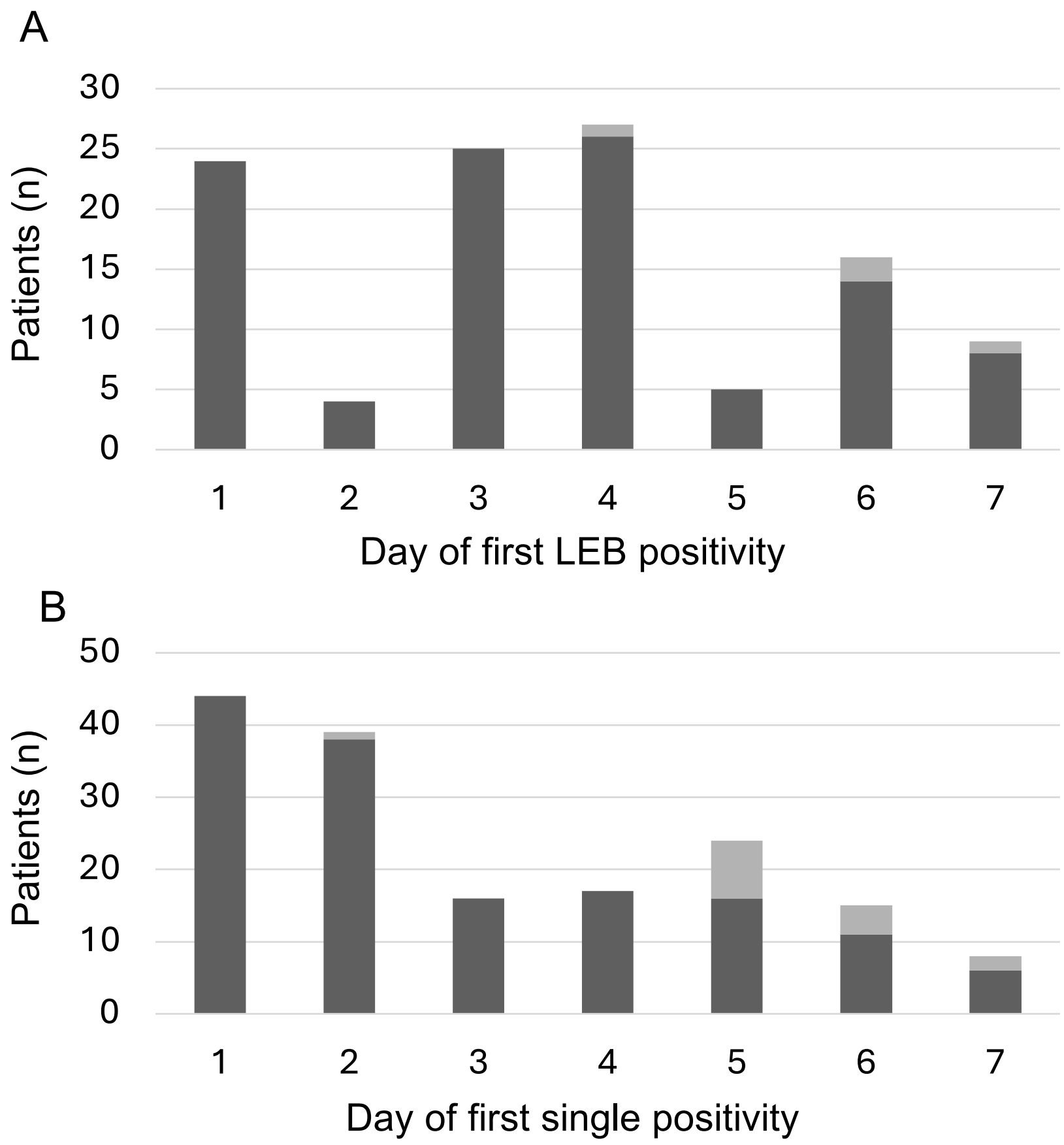

Figure S3. Distribution of CRRT initiation timing after ICU admission

Histogram showing the distribution of CRRT initiation timing during ICU stay among patients who received CRRT. Day 1 indicates CRRT initiation on the day of ICU admission. The category "≥8" represents CRRT initiation on ICU day 8 or later.

CRRT, continuous renal replacement therapy; ICU, intensive care unit.

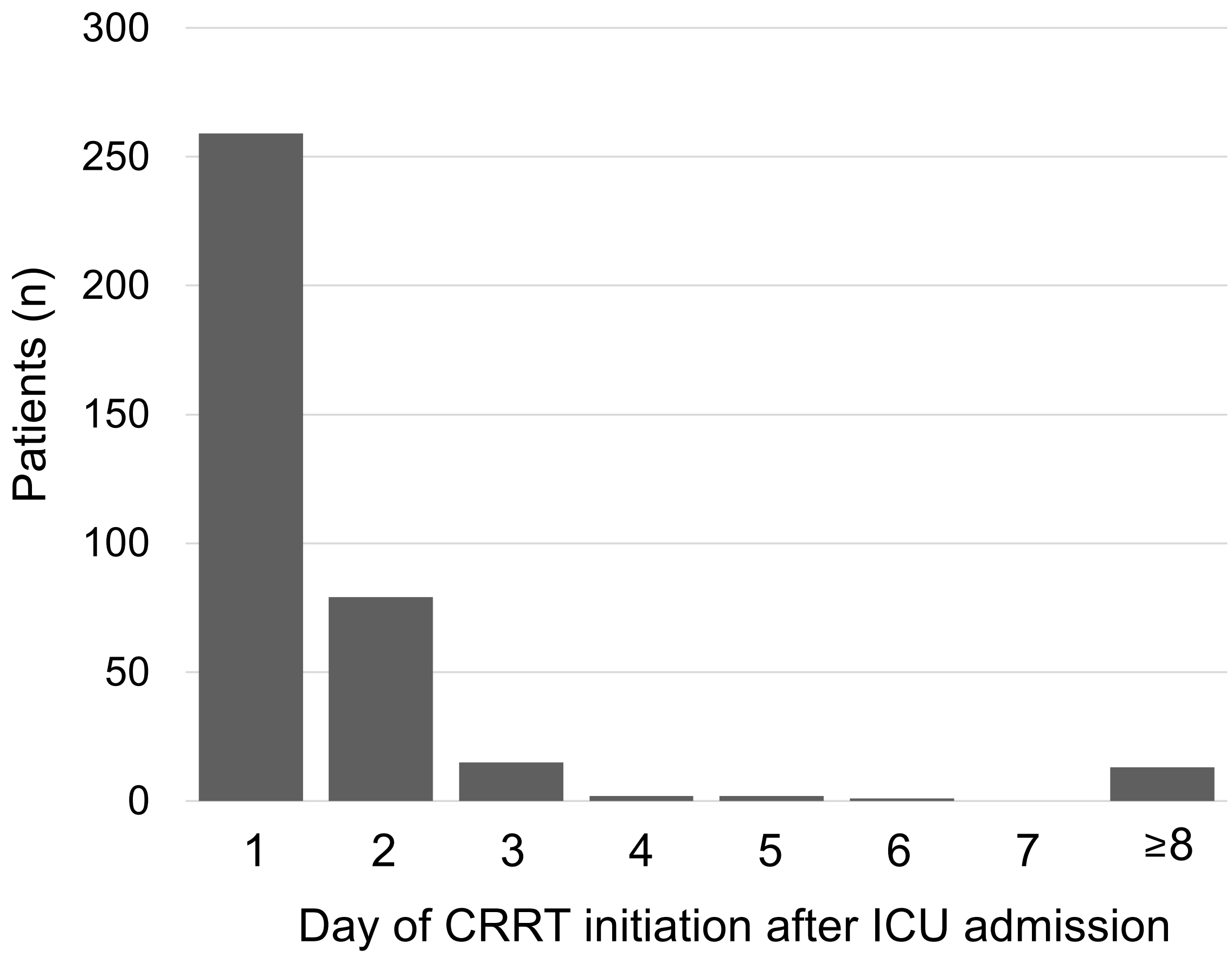

Figure S4. Kaplan–Meier 90-day survival from the day-7 landmark by five-group classification.

Patients who survived for at least 7 days after ICU admission were included in the study. Follow-up started 7 days after ICU admission (day-7 landmark) and continued until day 90 after ICU admission (censored at day 90). The groups were defined as described in Fig. 1. Tick marks indicate censoring. Numbers at risk are shown at days 7, 14, 30, 45, 60, 75, and 90.

IG, immature granulocytes; NRBC, nucleated red blood cells; LEB, leukoerythroblastosis.

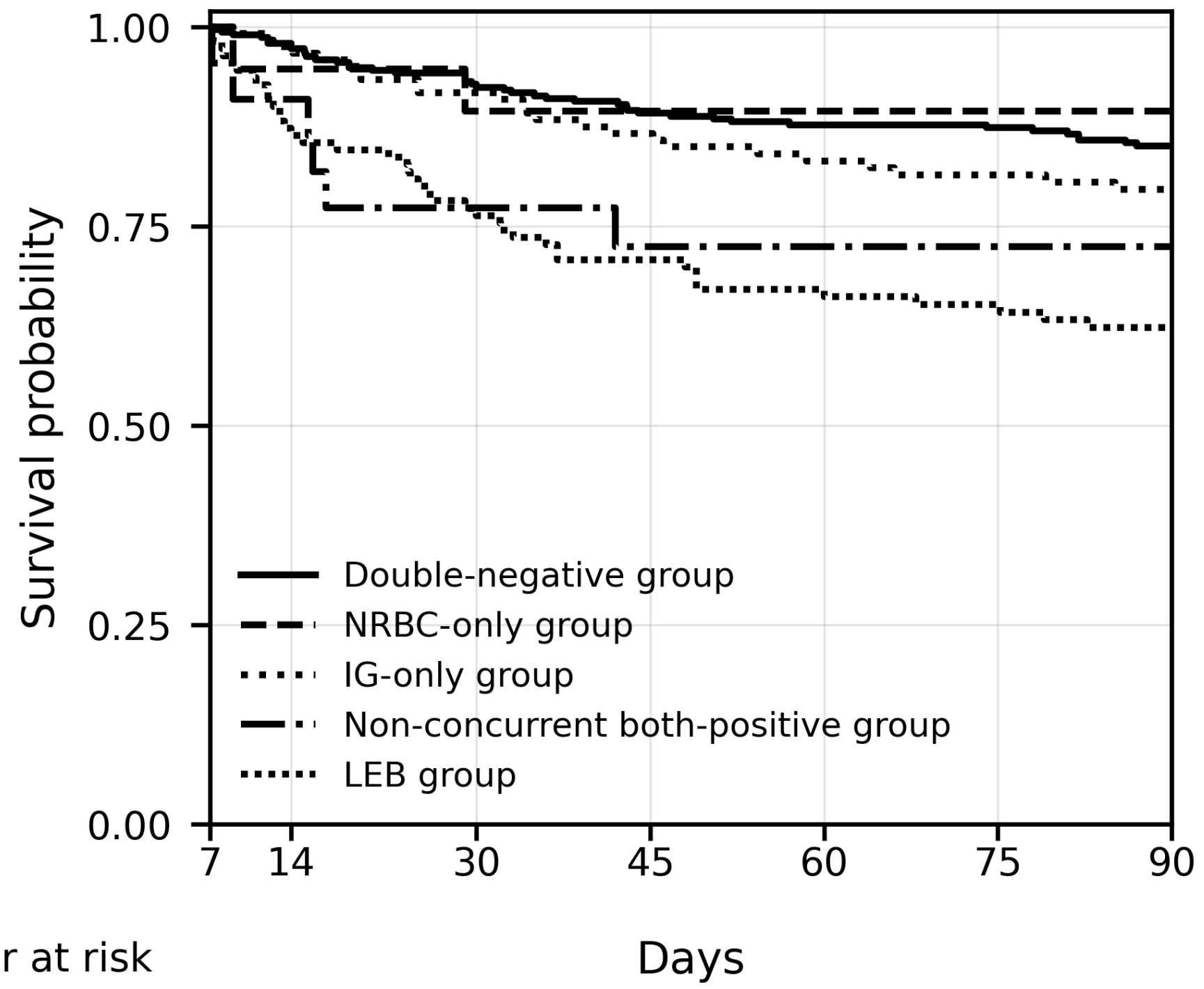

Number at risk

	7	14	30	45	60	75	90
Double-negative group	296	287	263	244	237	233	225
NRBC-only group	19	18	17	16	15	15	15
IG-only group	122	118	108	102	95	92	88
Non-concurrent both-positive group	22	20	16	15	15	15	15
LEB group	110	96	84	76	72	68	63
